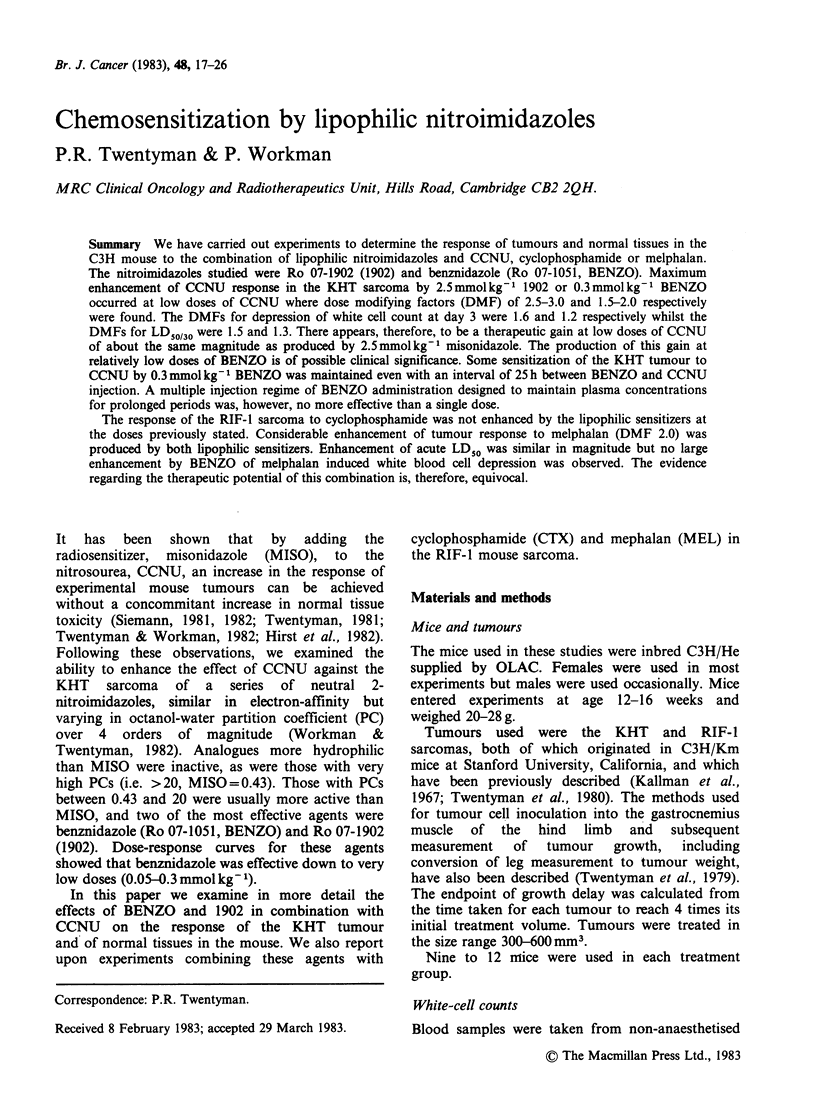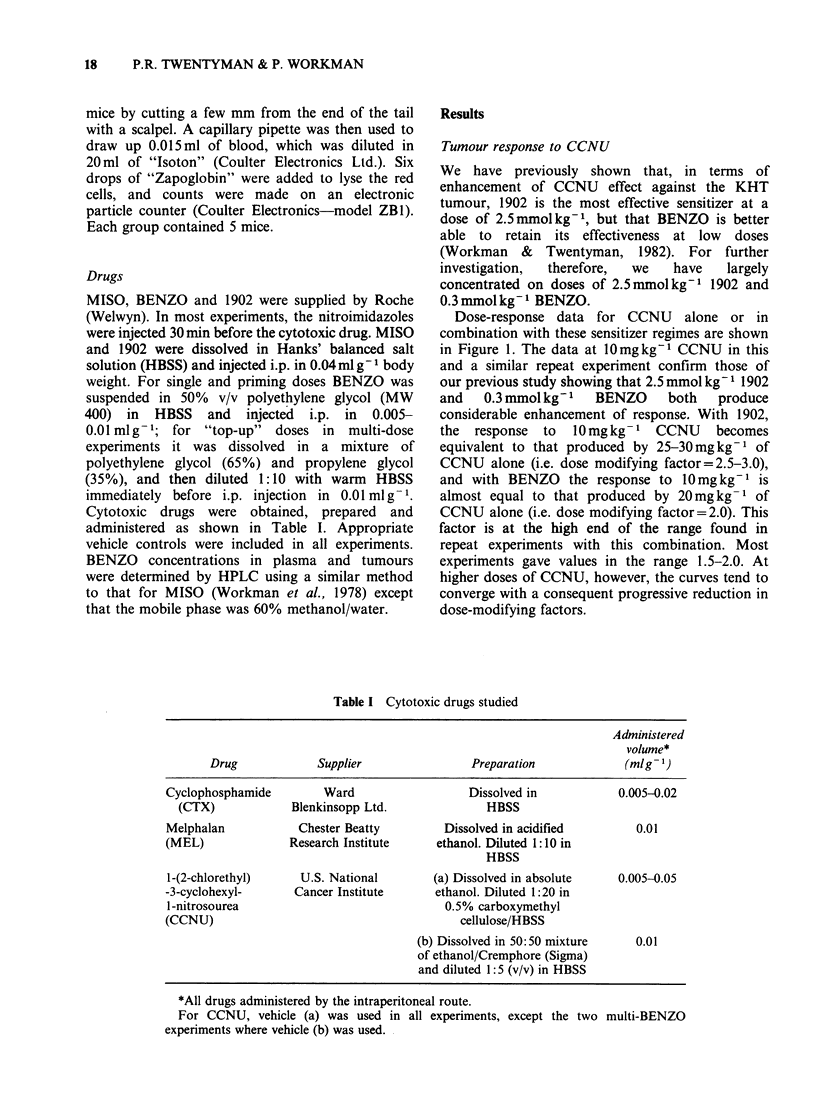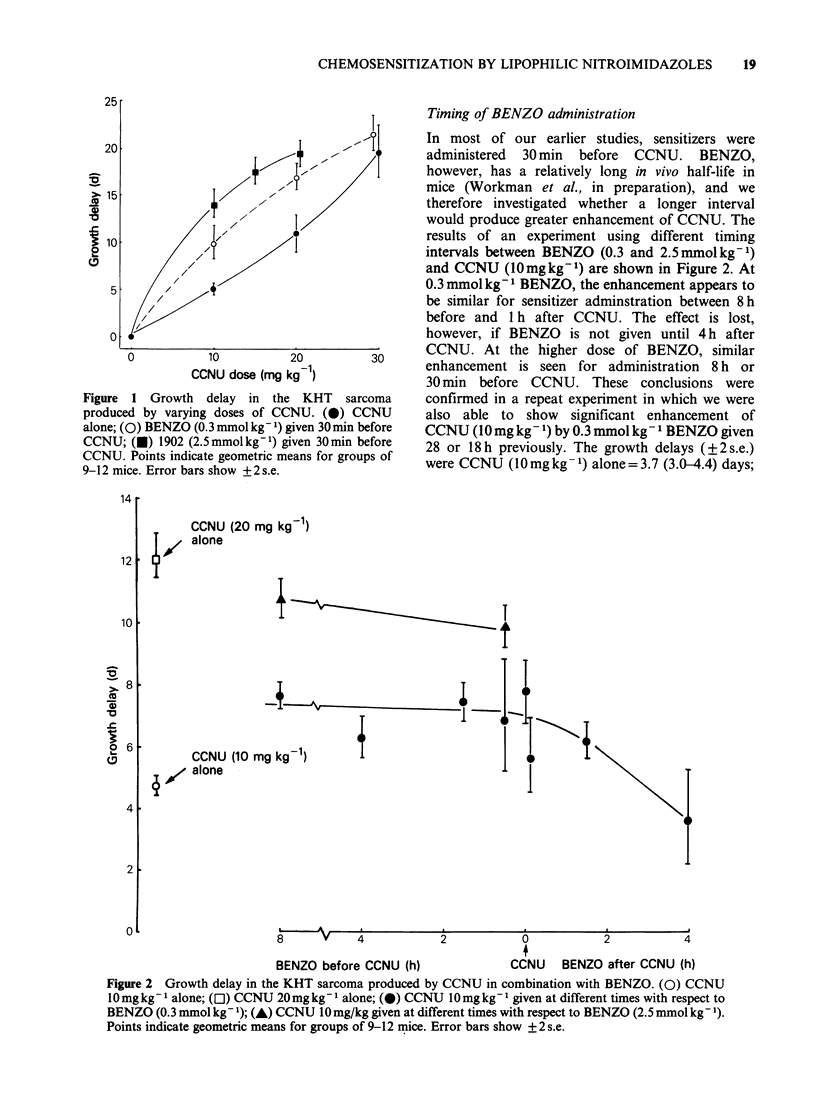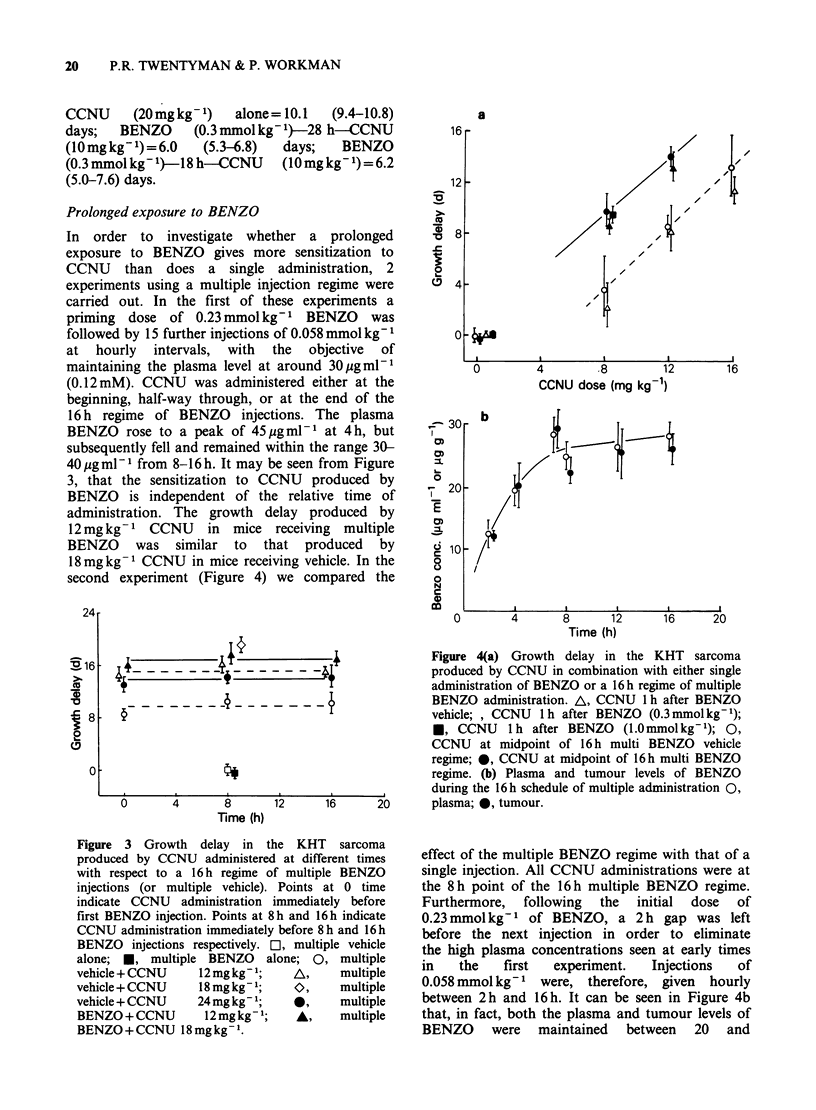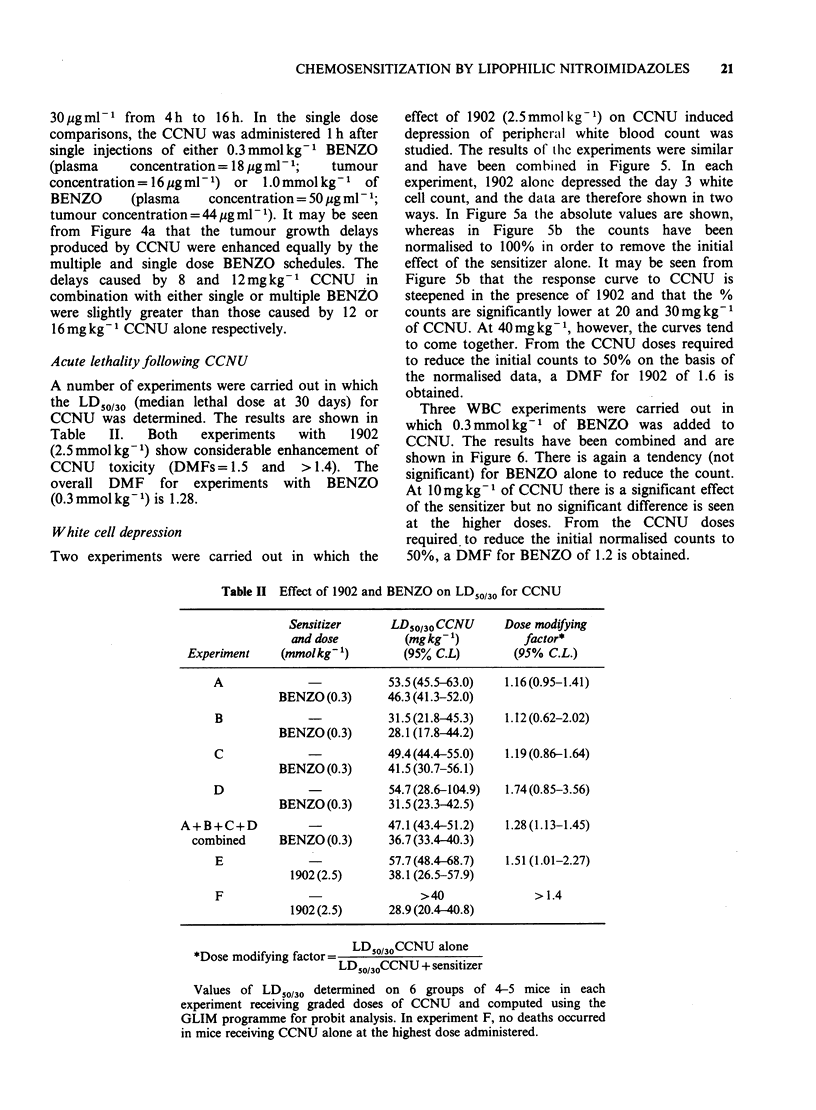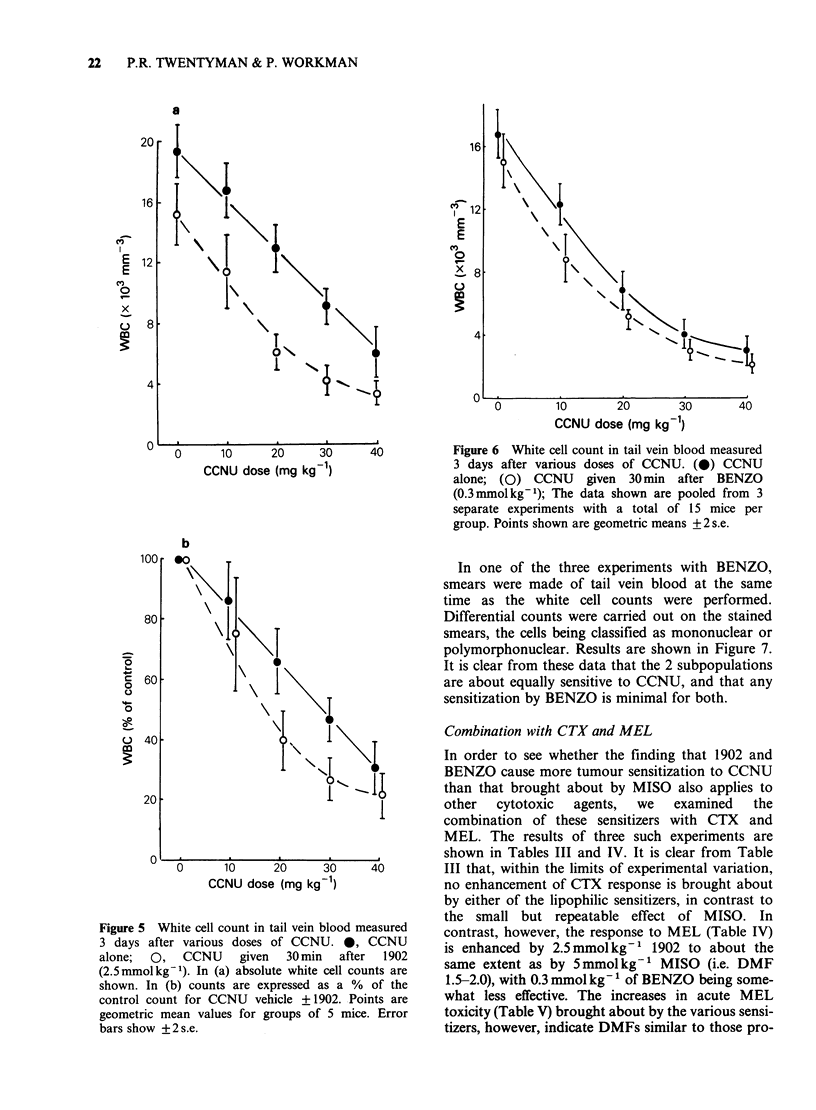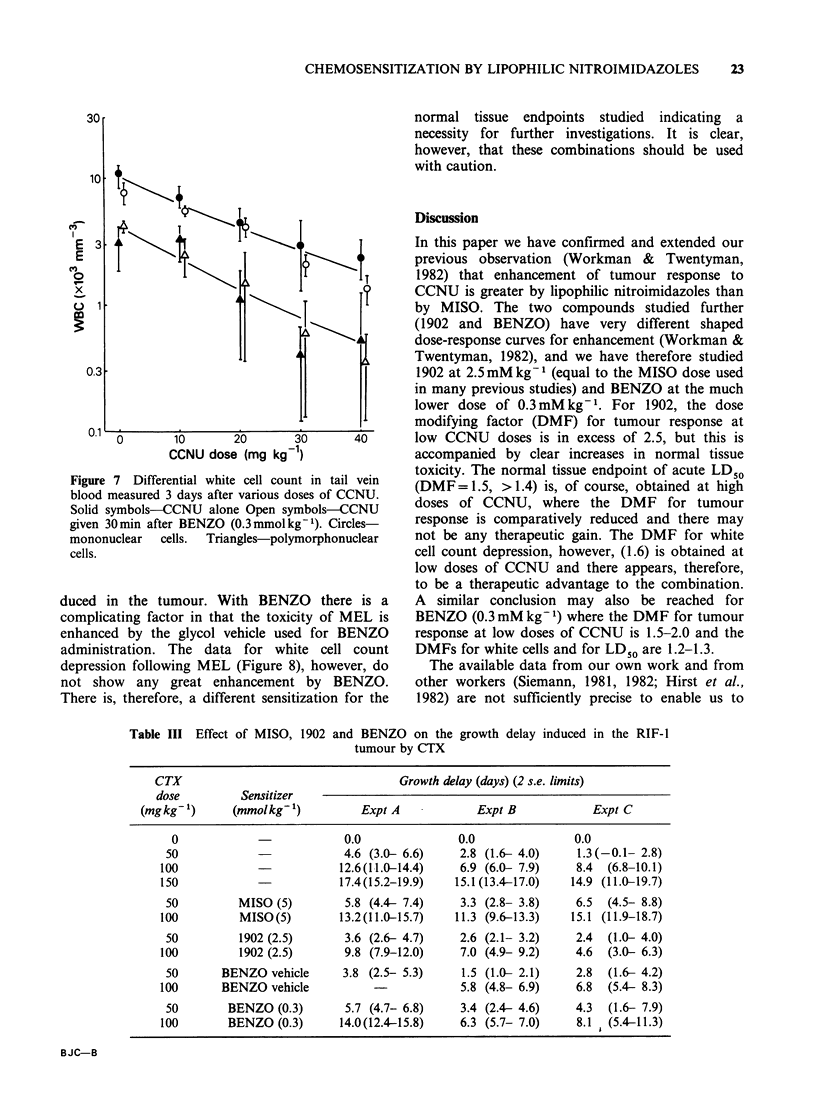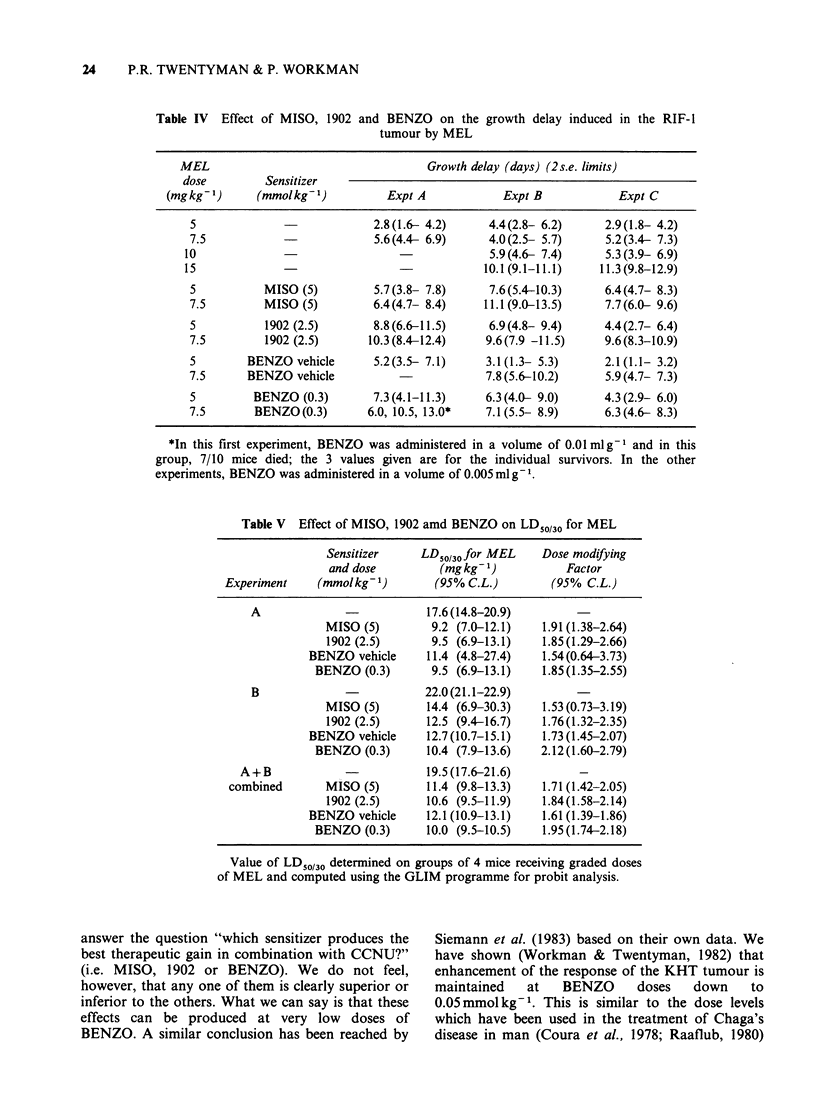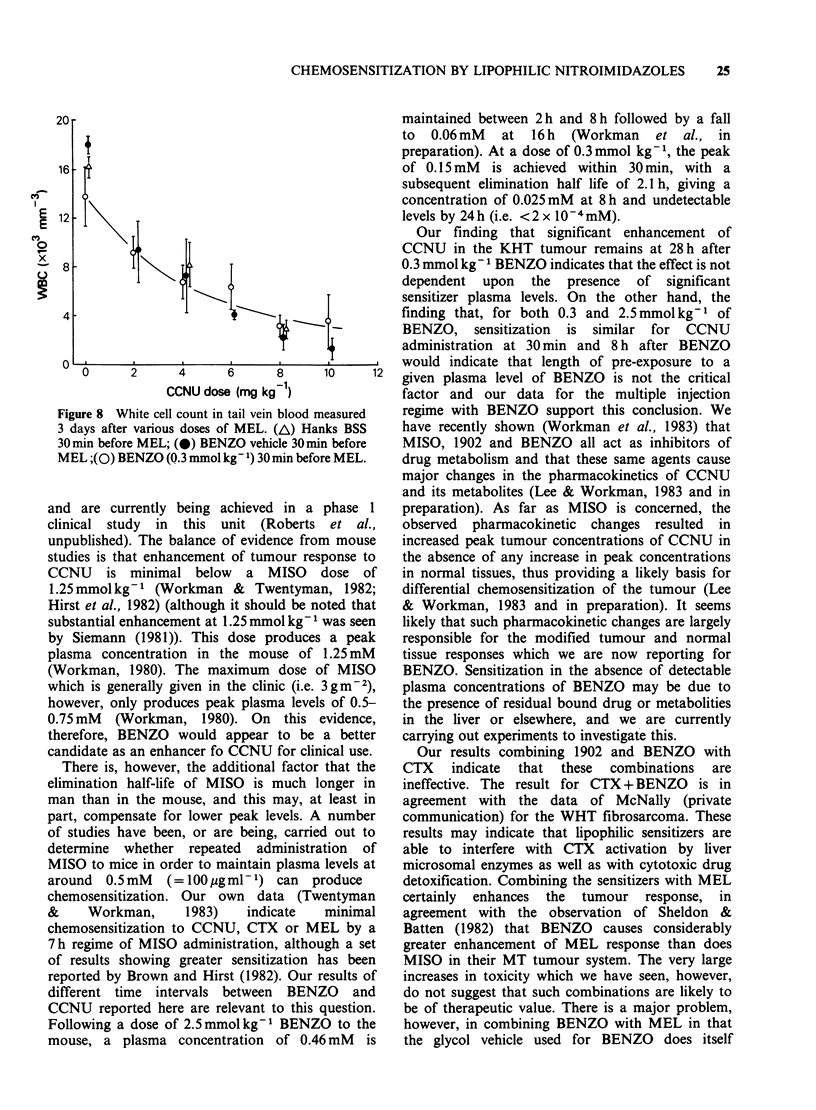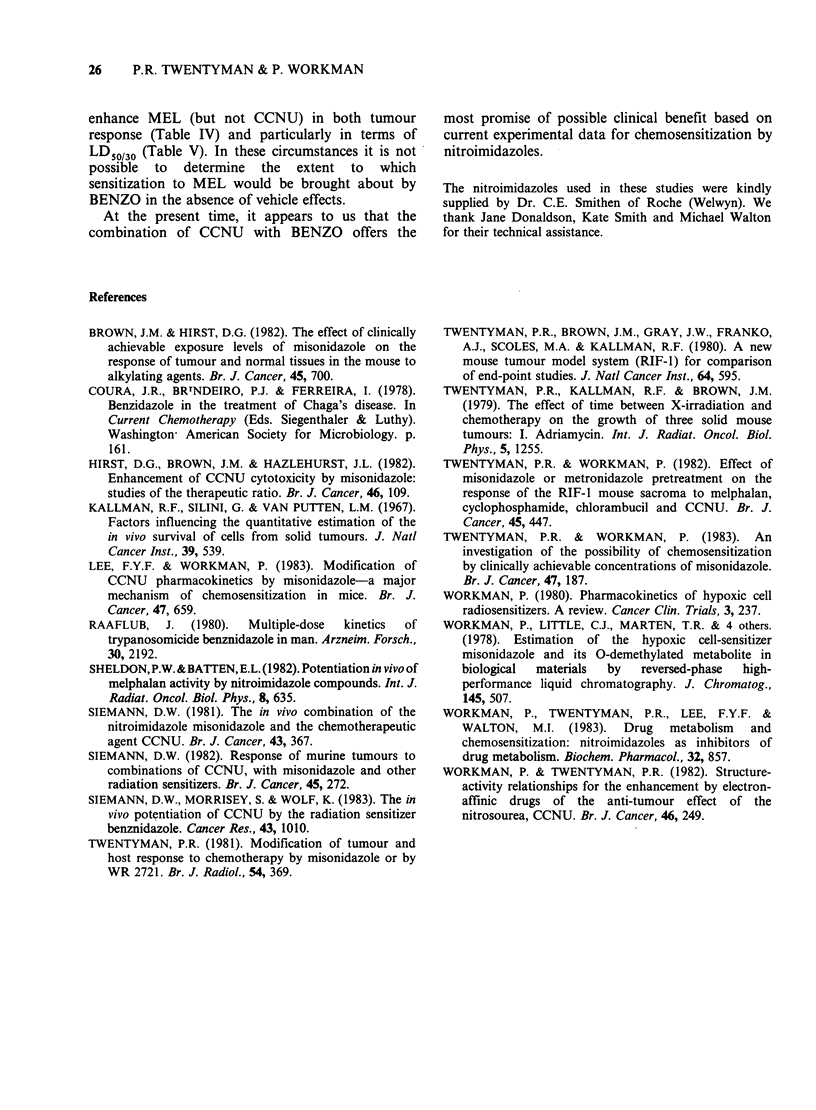# Chemosensitization by lipophilic nitroimidazoles.

**DOI:** 10.1038/bjc.1983.152

**Published:** 1983-07

**Authors:** P. R. Twentyman, P. Workman

## Abstract

We have carried out experiments to determine the response of tumours and normal tissues in the C3H mouse to the combination of lipophilic nitroimidazoles and CCNU, cyclophosphamide or melphalan. The nitroimidazoles studied were Ro 07-1902 (1902) and benznidazole (Ro 07-1051, BENZO). Maximum enhancement of CCNU response in the KHT sarcoma by 2.5 mmol kg-1 1902 or 0.3 mmol kg-1 BENZO occurred at low doses of CCNU where dose modifying factors (DMF) of 2.5-3.0 and 1.5-2.0 respectively were found. The DMFs for depression of white cell count at day 3 were 1.6 and 1.2 respectively whilst the DMFs for LD50/30 were 1.5 and 1.3. There appears, therefore, to be a therapeutic gain at low doses of CCNU of about the same magnitude as produced by 2.5 mmol kg-1 misonidazole. The production of this gain at relatively low doses of BENZO is of possible clinical significance. Some sensitization of the KHT tumour to CCNU by 0.3 mmol kg-1 BENZO was maintained even with an interval of 25 h between BENZO and CCNU injection. A multiple injection regime of BENZO administration designed to maintain plasma concentrations for prolonged periods was, however, no more effective than a single dose. The response of the RIF-1 sarcoma to cyclophosphamide was not enhanced by the lipophilic sensitizers at the doses previously stated. Considerable enhancement of tumour response to melphalan (DMF 2.0) was produced by both lipophilic sensitizers. Enhancement of acute LD50 was similar in magnitude but no large enhancement by BENZO of melphalan induced white blood cell depression was observed. The evidence regarding the therapeutic potential of this combination is, therefore, equivocal.


*Br. J. Cancer* (1983), **48**, 17–26

### Chemosensitization by lipophilic nitroimidazoles

P.R. Twentyman & P. Workman

*MRC Clinical Oncology and Radiotherapeutics Unit, Hills Road, Cambridge CB2 2QH.*

**Summary** We have carried out experiments to determine the response of tumours and normal tissues in the C3H mouse to the combination of lipophilic nitroimidazoles and CCNU, cyclophosphamide or melphalan. The nitroimidazoles studied were Ro 07-1902 (1902) and benznidazole (Ro 07-1051, BENZO). Maximum enhancement of CCNU response in the KHT sarcoma by 2.5 mmol kg$^{-1}$ 1902 or 0.3 mmol kg$^{-1}$ BENZO occurred at low doses of CCNU where dose modifying factors (DMF) of 2.5–3.0 and 1.5–2.0 respectively were found. The DMFs for depression of white cell count at day 3 were 1.6 and 1.2 respectively whilst the DMFs for $LD_{50/30}$ were 1.5 and 1.3. There appears, therefore, to be a therapeutic gain at low doses of CCNU of about the same magnitude as produced by 2.5 mmol kg$^{-1}$ misonidazole. The production of this gain at relatively low doses of BENZO is of possible clinical significance. Some sensitization of the KHT tumour to CCNU by 0.3 mmol kg$^{-1}$ BENZO was maintained even with an interval of 25 h between BENZO and CCNU injection. A multiple injection regime of BENZO administration designed to maintain plasma concentrations for prolonged periods was, however, no more effective than a single dose.

The response of the RIF-1 sarcoma to cyclophosphamide was not enhanced by the lipophilic sensitizers at the doses previously stated. Considerable enhancement of tumour response to melphalan (DMF 2.0) was produced by both lipophilic sensitizers. Enhancement of acute $LD_{50}$ was similar in magnitude but no large enhancement by BENZO of melphalan induced white blood cell depression was observed. The evidence regarding the therapeutic potential of this combination is, therefore, equivocal.

It has been shown that by adding the radiosensitizer, misonidazole (MISO), to the nitrosourea, CCNU, an increase in the response of experimental mouse tumours can be achieved without a concommitant increase in normal tissue toxicity (Siemann, 1981, 1982; Twentyman, 1981; Twentyman & Workman, 1982; Hirst *et al.*, 1982). Following these observations, we examined the ability to enhance the effect of CCNU against the KHT sarcoma of a series of neutral 2-nitroimidazoles, similar in electron-affinity but varying in octanol-water partition coefficient (PC) over 4 orders of magnitude (Workman & Twentyman, 1982). Analogues more hydrophilic than MISO were inactive, as were those with very high PCs (i.e. >20, MISO = 0.43). Those with PCs between 0.43 and 20 were usually more active than MISO, and two of the most effective agents were benznidazole (Ro 07-1051, BENZO) and Ro 07-1902 (1902). Dose-response curves for these agents showed that benznidazole was effective down to very low doses (0.05–0.3 mmol kg$^{-1}$).

In this paper we examine in more detail the effects of BENZO and 1902 in combination with CCNU on the response of the KHT tumour and of normal tissues in the mouse. We also report upon experiments combining these agents with cyclophosphamide (CTX) and mephalan (MEL) in the RIF-1 mouse sarcoma.

Correspondence: P.R. Twentyman.

Received 8 February 1983; accepted 29 March 1983.

### Materials and methods

### *Mice and tumours*

The mice used in these studies were inbred C3H/He supplied by OLAC. Females were used in most experiments but males were used occasionally. Mice entered experiments at age 12–16 weeks and weighed 20–28 g.

Tumours used were the KHT and RIF-1 sarcomas, both of which originated in C3H/Km mice at Stanford University, California, and which have been previously described (Kallman *et al.*, 1967; Twentyman *et al.*, 1980). The methods used for tumour cell inoculation into the gastrocnemius muscle of the hind limb and subsequent measurement of tumour growth, including conversion of leg measurement to tumour weight, have also been described (Twentyman *et al.*, 1979). The endpoint of growth delay was calculated from the time taken for each tumour to reach 4 times its initial treatment volume. Tumours were treated in the size range 300–600 mm$^3$.

Nine to 12 mice were used in each treatment group.

### *White-cell counts*

Blood samples were taken from non-anaesthetised

© The Macmillan Press Ltd., 1983

mice by cutting a few mm from the end of the tail with a scalpel. A capillary pipette was then used to draw up 0.015 ml of blood, which was diluted in 20 ml of "Isoton" (Coulter Electronics Ltd.). Six drops of "Zapoglobin" were added to lyse the red cells, and counts were made on an electronic particle counter (Coulter Electronics—model ZB1). Each group contained 5 mice.

### *Drugs*

MISO, BENZO and 1902 were supplied by Roche (Welwyn). In most experiments, the nitroimidazoles were injected 30 min before the cytotoxic drug. MISO and 1902 were dissolved in Hanks' balanced salt solution (HBSS) and injected i.p. in 0.04 ml $g^{-1}$ body weight. For single and priming doses BENZO was suspended in 50% v/v polyethylene glycol (MW 400) in HBSS and injected i.p. in 0.005–0.01 ml $g^{-1}$; for "top-up" doses in multi-dose experiments it was dissolved in a mixture of polyethylene glycol (65%) and propylene glycol (35%), and then diluted 1:10 with warm HBSS immediately before i.p. injection in 0.01 ml $g^{-1}$. Cytotoxic drugs were obtained, prepared and administered as shown in Table I. Appropriate vehicle controls were included in all experiments. BENZO concentrations in plasma and tumours were determined by HPLC using a similar method to that for MISO (Workman *et al.*, 1978) except that the mobile phase was 60% methanol/water.

### Results

### *Tumour response to CCNU*

We have previously shown that, in terms of enhancement of CCNU effect against the KHT tumour, 1902 is the most effective sensitizer at a dose of 2.5 mmol $kg^{-1}$, but that BENZO is better able to retain its effectiveness at low doses (Workman & Twentyman, 1982). For further investigation, therefore, we have largely concentrated on doses of 2.5 mmol $kg^{-1}$ 1902 and 0.3 mmol $kg^{-1}$ BENZO.

Dose-response data for CCNU alone or in combination with these sensitizer regimes are shown in Figure 1. The data at 10 mg $kg^{-1}$ CCNU in this and a similar repeat experiment confirm those of our previous study showing that 2.5 mmol $kg^{-1}$ 1902 and 0.3 mmol $kg^{-1}$ BENZO both produce considerable enhancement of response. With 1902, the response to 10 mg $kg^{-1}$ CCNU becomes equivalent to that produced by 25–30 mg $kg^{-1}$ of CCNU alone (i.e. dose modifying factor = 2.5–3.0), and with BENZO the response to 10 mg $kg^{-1}$ is almost equal to that produced by 20 mg $kg^{-1}$ of CCNU alone (i.e. dose modifying factor = 2.0). This factor is at the high end of the range found in repeat experiments with this combination. Most experiments gave values in the range 1.5–2.0. At higher doses of CCNU, however, the curves tend to converge with a consequent progressive reduction in dose-modifying factors.

**Table I** Cytotoxic drugs studied

| *Drug* | *Supplier* | *Preparation* | *Administered volume* ($ml\,g^{-1}$)* |
|---|---|---|---|
| Cyclophosphamide (CTX) | Ward Blenkinsopp Ltd. | Dissolved in HBSS | 0.005–0.02 |
| Melphalan (MEL) | Chester Beatty Research Institute | Dissolved in acidified ethanol. Diluted 1:10 in HBSS | 0.01 |
| 1-(2-chlorethyl) -3-cyclohexyl-1-nitrosourea (CCNU) | U.S. National Cancer Institute | (a) Dissolved in absolute ethanol. Diluted 1:20 in 0.5% carboxymethyl cellulose/HBSS | 0.005–0.05 |
| | | (b) Dissolved in 50:50 mixture of ethanol/Cremphore (Sigma) and diluted 1:5 (v/v) in HBSS | 0.01 |

*All drugs administered by the intraperitoneal route.

For CCNU, vehicle (a) was used in all experiments, except the two multi-BENZO experiments where vehicle (b) was used.

**Figure 1** Growth delay in the KHT sarcoma produced by varying doses of CCNU. (●) CCNU alone; (○) BENZO (0.3 mmol kg$^{-1}$) given 30 min before CCNU; (■) 1902 (2.5 mmol kg$^{-1}$) given 30 min before CCNU. Points indicate geometric means for groups of 9–12 mice. Error bars show ±2 s.e.

*Timing of BENZO administration*

In most of our earlier studies, sensitizers were administered 30 min before CCNU. BENZO, however, has a relatively long *in vivo* half-life in mice (Workman *et al.*, in preparation), and we therefore investigated whether a longer interval would produce greater enhancement of CCNU. The results of an experiment using different timing intervals between BENZO (0.3 and 2.5 mmol kg$^{-1}$) and CCNU (10 mg kg$^{-1}$) are shown in Figure 2. At 0.3 mmol kg$^{-1}$ BENZO, the enhancement appears to be similar for sensitizer adminstration between 8 h before and 1 h after CCNU. The effect is lost, however, if BENZO is not given until 4 h after CCNU. At the higher dose of BENZO, similar enhancement is seen for administration 8 h or 30 min before CCNU. These conclusions were confirmed in a repeat experiment in which we were also able to show significant enhancement of CCNU (10 mg kg$^{-1}$) by 0.3 mmol kg$^{-1}$ BENZO given 28 or 18 h previously. The growth delays (±2 s.e.) were CCNU (10 mg kg$^{-1}$) alone = 3.7 (3.0–4.4) days;

**Figure 2** Growth delay in the KHT sarcoma produced by CCNU in combination with BENZO. (○) CCNU 10 mg kg$^{-1}$ alone; (□) CCNU 20 mg kg$^{-1}$ alone; (●) CCNU 10 mg kg$^{-1}$ given at different times with respect to BENZO (0.3 mmol kg$^{-1}$); (▲) CCNU 10 mg/kg given at different times with respect to BENZO (2.5 mmol kg$^{-1}$). Points indicate geometric means for groups of 9–12 mice. Error bars show ±2 s.e.

CCNU ($20\,mg\,kg^{-1}$) alone = 10.1 (9.4–10.8) days; BENZO ($0.3\,mmol\,kg^{-1}$)—28 h—CCNU ($10\,mg\,kg^{-1}$) = 6.0 (5.3–6.8) days; BENZO ($0.3\,mmol\,kg^{-1}$)—18 h—CCNU ($10\,mg\,kg^{-1}$) = 6.2 (5.0–7.6) days.

*Prolonged exposure to BENZO*

In order to investigate whether a prolonged exposure to BENZO gives more sensitization to CCNU than does a single administration, 2 experiments using a multiple injection regime were carried out. In the first of these experiments a priming dose of $0.23\,mmol\,kg^{-1}$ BENZO was followed by 15 further injections of $0.058\,mmol\,kg^{-1}$ at hourly intervals, with the objective of maintaining the plasma level at around $30\,\mu g\,ml^{-1}$ (0.12 mM). CCNU was administered either at the beginning, half-way through, or at the end of the 16 h regime of BENZO injections. The plasma BENZO rose to a peak of $45\,\mu g\,ml^{-1}$ at 4 h, but subsequently fell and remained within the range 30–40 $\mu g\,ml^{-1}$ from 8–16 h. It may be seen from Figure 3, that the sensitization to CCNU produced by BENZO is independent of the relative time of administration. The growth delay produced by $12\,mg\,kg^{-1}$ CCNU in mice receiving multiple BENZO was similar to that produced by $18\,mg\,kg^{-1}$ CCNU in mice receiving vehicle. In the second experiment (Figure 4) we compared the effect of the multiple BENZO regime with that of a single injection. All CCNU administrations were at the 8 h point of the 16 h multiple BENZO regime. Furthermore, following the initial dose of $0.23\,mmol\,kg^{-1}$ of BENZO, a 2 h gap was left before the next injection in order to eliminate the high plasma concentrations seen at early times in the first experiment. Injections of $0.058\,mmol\,kg^{-1}$ were, therefore, given hourly between 2 h and 16 h. It can be seen in Figure 4b that, in fact, both the plasma and tumour levels of BENZO were maintained between 20 and

**Figure 3** Growth delay in the KHT sarcoma produced by CCNU administered at different times with respect to a 16 h regime of multiple BENZO injections (or multiple vehicle). Points at 0 time indicate CCNU administration immediately before first BENZO injection. Points at 8 h and 16 h indicate CCNU administration immediately before 8 h and 16 h BENZO injections respectively. □, multiple vehicle alone; ■, multiple BENZO alone; ○, multiple vehicle + CCNU $12\,mg\,kg^{-1}$; △, multiple vehicle + CCNU $18\,mg\,kg^{-1}$; ◇, multiple vehicle + CCNU $24\,mg\,kg^{-1}$; ●, multiple BENZO + CCNU $12\,mg\,kg^{-1}$; ▲, multiple BENZO + CCNU $18\,mg\,kg^{-1}$.

**Figure 4(a)** Growth delay in the KHT sarcoma produced by CCNU in combination with either single administration of BENZO or a 16 h regime of multiple BENZO administration. △, CCNU 1 h after BENZO vehicle; , CCNU 1 h after BENZO ($0.3\,mmol\,kg^{-1}$); ■, CCNU 1 h after BENZO ($1.0\,mmol\,kg^{-1}$); ○, CCNU at midpoint of 16 h multi BENZO vehicle regime; ●, CCNU at midpoint of 16 h multi BENZO regime. **(b)** Plasma and tumour levels of BENZO during the 16 h schedule of multiple administration ○, plasma; ●, tumour.

$30\,\mu g\,ml^{-1}$ from 4h to 16h. In the single dose comparisons, the CCNU was administered 1 h after single injections of either $0.3\,mmol\,kg^{-1}$ BENZO (plasma concentration $= 18\,\mu g\,ml^{-1}$; tumour concentration $= 16\,\mu g\,ml^{-1}$) or $1.0\,mmol\,kg^{-1}$ of BENZO (plasma concentration $= 50\,\mu g\,ml^{-1}$; tumour concentration $= 44\,\mu g\,ml^{-1}$). It may be seen from Figure 4a that the tumour growth delays produced by CCNU were enhanced equally by the multiple and single dose BENZO schedules. The delays caused by 8 and $12\,mg\,kg^{-1}$ CCNU in combination with either single or multiple BENZO were slightly greater than those caused by 12 or $16\,mg\,kg^{-1}$ CCNU alone respectively.

*Acute lethality following CCNU*

A number of experiments were carried out in which the $LD_{50/30}$ (median lethal dose at 30 days) for CCNU was determined. The results are shown in Table II. Both experiments with 1902 ($2.5\,mmol\,kg^{-1}$) show considerable enhancement of CCNU toxicity (DMFs = 1.5 and > 1.4). The overall DMF for experiments with BENZO ($0.3\,mmol\,kg^{-1}$) is 1.28.

*White cell depression*

Two experiments were carried out in which the effect of 1902 ($2.5\,mmol\,kg^{-1}$) on CCNU induced depression of peripheral white blood count was studied. The results of the experiments were similar and have been combined in Figure 5. In each experiment, 1902 alone depressed the day 3 white cell count, and the data are therefore shown in two ways. In Figure 5a the absolute values are shown, whereas in Figure 5b the counts have been normalised to 100% in order to remove the initial effect of the sensitizer alone. It may be seen from Figure 5b that the response curve to CCNU is steepened in the presence of 1902 and that the % counts are significantly lower at 20 and $30\,mg\,kg^{-1}$ of CCNU. At $40\,mg\,kg^{-1}$, however, the curves tend to come together. From the CCNU doses required to reduce the initial counts to 50% on the basis of the normalised data, a DMF for 1902 of 1.6 is obtained.

Three WBC experiments were carried out in which $0.3\,mmol\,kg^{-1}$ of BENZO was added to CCNU. The results have been combined and are shown in Figure 6. There is again a tendency (not significant) for BENZO alone to reduce the count. At $10\,mg\,kg^{-1}$ of CCNU there is a significant effect of the sensitizer but no significant difference is seen at the higher doses. From the CCNU doses required to reduce the initial normalised counts to 50%, a DMF for BENZO of 1.2 is obtained.

**Table II** Effect of 1902 and BENZO on $LD_{50/30}$ for CCNU

| *Experiment* | *Sensitizer and dose* ($mmol\,kg^{-1}$) | $LD_{50/30}$ *CCNU* ($mg\,kg^{-1}$) (95% C.L) | *Dose modifying factor** (95% C.L.) |
|---|---|---|---|
| A | — | 53.5 (45.5–63.0) | 1.16 (0.95–1.41) |
| | BENZO (0.3) | 46.3 (41.3–52.0) | |
| B | — | 31.5 (21.8–45.3) | 1.12 (0.62–2.02) |
| | BENZO (0.3) | 28.1 (17.8–44.2) | |
| C | — | 49.4 (44.4–55.0) | 1.19 (0.86–1.64) |
| | BENZO (0.3) | 41.5 (30.7–56.1) | |
| D | — | 54.7 (28.6–104.9) | 1.74 (0.85–3.56) |
| | BENZO (0.3) | 31.5 (23.3–42.5) | |
| A+B+C+D | — | 47.1 (43.4–51.2) | 1.28 (1.13–1.45) |
| combined | BENZO (0.3) | 36.7 (33.4–40.3) | |
| E | — | 57.7 (48.4–68.7) | 1.51 (1.01–2.27) |
| | 1902 (2.5) | 38.1 (26.5–57.9) | |
| F | — | > 40 | > 1.4 |
| | 1902 (2.5) | 28.9 (20.4–40.8) | |

$$*\text{Dose modifying factor} = \frac{LD_{50/30}\text{CCNU alone}}{LD_{50/30}\text{CCNU} + \text{sensitizer}}$$

Values of $LD_{50/30}$ determined on 6 groups of 4–5 mice in each experiment receiving graded doses of CCNU and computed using the GLIM programme for probit analysis. In experiment F, no deaths occurred in mice receiving CCNU alone at the highest dose administered.

**Figure 5** White cell count in tail vein blood measured 3 days after various doses of CCNU. ●, CCNU alone; ○, CCNU given 30 min after 1902 (2.5 mmol $kg^{-1}$). In (a) absolute white cell counts are shown. In (b) counts are expressed as a % of the control count for CCNU vehicle ±1902. Points are geometric mean values for groups of 5 mice. Error bars show ±2 s.e.

**Figure 6** White cell count in tail vein blood measured 3 days after various doses of CCNU. (●) CCNU alone; (○) CCNU given 30 min after BENZO (0.3 mmol $kg^{-1}$); The data shown are pooled from 3 separate experiments with a total of 15 mice per group. Points shown are geometric means ±2 s.e.

In one of the three experiments with BENZO, smears were made of tail vein blood at the same time as the white cell counts were performed. Differential counts were carried out on the stained smears, the cells being classified as mononuclear or polymorphonuclear. Results are shown in Figure 7. It is clear from these data that the 2 subpopulations are about equally sensitive to CCNU, and that any sensitization by BENZO is minimal for both.

*Combination with CTX and MEL*

In order to see whether the finding that 1902 and BENZO cause more tumour sensitization to CCNU than that brought about by MISO also applies to other cytotoxic agents, we examined the combination of these sensitizers with CTX and MEL. The results of three such experiments are shown in Tables III and IV. It is clear from Table III that, within the limits of experimental variation, no enhancement of CTX response is brought about by either of the lipophilic sensitizers, in contrast to the small but repeatable effect of MISO. In contrast, however, the response to MEL (Table IV) is enhanced by 2.5 mmol $kg^{-1}$ 1902 to about the same extent as by 5 mmol $kg^{-1}$ MISO (i.e. DMF 1.5–2.0), with 0.3 mmol $kg^{-1}$ of BENZO being somewhat less effective. The increases in acute MEL toxicity (Table V) brought about by the various sensitizers, however, indicate DMFs similar to those pro-

**Figure 7** Differential white cell count in tail vein blood measured 3 days after various doses of CCNU. Solid symbols—CCNU alone Open symbols—CCNU given 30 min after BENZO (0.3 mmol kg$^{-1}$). Circles—mononuclear cells. Triangles—polymorphonuclear cells.

duced in the tumour. With BENZO there is a complicating factor in that the toxicity of MEL is enhanced by the glycol vehicle used for BENZO administration. The data for white cell count depression following MEL (Figure 8), however, do not show any great enhancement by BENZO. There is, therefore, a different sensitization for the normal tissue endpoints studied indicating a necessity for further investigations. It is clear, however, that these combinations should be used with caution.

### Discussion

In this paper we have confirmed and extended our previous observation (Workman & Twentyman, 1982) that enhancement of tumour response to CCNU is greater by lipophilic nitroimidazoles than by MISO. The two compounds studied further (1902 and BENZO) have very different shaped dose-response curves for enhancement (Workman & Twentyman, 1982), and we have therefore studied 1902 at 2.5 mM kg$^{-1}$ (equal to the MISO dose used in many previous studies) and BENZO at the much lower dose of 0.3 mM kg$^{-1}$. For 1902, the dose modifying factor (DMF) for tumour response at low CCNU doses is in excess of 2.5, but this is accompanied by clear increases in normal tissue toxicity. The normal tissue endpoint of acute $LD_{50}$ (DMF = 1.5, > 1.4) is, of course, obtained at high doses of CCNU, where the DMF for tumour response is comparatively reduced and there may not be any therapeutic gain. The DMF for white cell count depression, however, (1.6) is obtained at low doses of CCNU and there appears, therefore, to be a therapeutic advantage to the combination. A similar conclusion may also be reached for BENZO (0.3 mM kg$^{-1}$) where the DMF for tumour response at low doses of CCNU is 1.5–2.0 and the DMFs for white cells and for $LD_{50}$ are 1.2–1.3.

The available data from our own work and from other workers (Siemann, 1981, 1982; Hirst *et al.*, 1982) are not sufficiently precise to enable us to

**Table III** Effect of MISO, 1902 and BENZO on the growth delay induced in the RIF-1 tumour by CTX

| *CTX dose (mg kg$^{-1}$)* | *Sensitizer (mmol kg$^{-1}$)* | *Growth delay (days) (2 s.e. limits)* Expt A | Expt B | Expt C |
|---|---|---|---|---|
| 0 | — | 0.0 | 0.0 | 0.0 |
| 50 | — | 4.6 (3.0– 6.6) | 2.8 (1.6– 4.0) | 1.3 (−0.1– 2.8) |
| 100 | — | 12.6 (11.0–14.4) | 6.9 (6.0– 7.9) | 8.4 (6.8–10.1) |
| 150 | — | 17.4 (15.2–19.9) | 15.1 (13.4–17.0) | 14.9 (11.0–19.7) |
| 50 | MISO (5) | 5.8 (4.4– 7.4) | 3.3 (2.8– 3.8) | 6.5 (4.5– 8.8) |
| 100 | MISO (5) | 13.2 (11.0–15.7) | 11.3 (9.6–13.3) | 15.1 (11.9–18.7) |
| 50 | 1902 (2.5) | 3.6 (2.6– 4.7) | 2.6 (2.1– 3.2) | 2.4 (1.0– 4.0) |
| 100 | 1902 (2.5) | 9.8 (7.9–12.0) | 7.0 (4.9– 9.2) | 4.6 (3.0– 6.3) |
| 50 | BENZO vehicle | 3.8 (2.5– 5.3) | 1.5 (1.0– 2.1) | 2.8 (1.6– 4.2) |
| 100 | BENZO vehicle | — | 5.8 (4.8– 6.9) | 6.8 (5.4– 8.3) |
| 50 | BENZO (0.3) | 5.7 (4.7– 6.8) | 3.4 (2.4– 4.6) | 4.3 (1.6– 7.9) |
| 100 | BENZO (0.3) | 14.0 (12.4–15.8) | 6.3 (5.7– 7.0) | 8.1 (5.4–11.3) |

**Table IV** Effect of MISO, 1902 and BENZO on the growth delay induced in the RIF-1 tumour by MEL

| *MEL dose* ($mg\,kg^{-1}$) | *Sensitizer* ($mmol\,kg^{-1}$) | *Growth delay (days) (2 s.e. limits)* | | |
|---|---|---|---|---|
| | | *Expt A* | *Expt B* | *Expt C* |
| 5 | — | 2.8 (1.6– 4.2) | 4.4 (2.8– 6.2) | 2.9 (1.8– 4.2) |
| 7.5 | — | 5.6 (4.4– 6.9) | 4.0 (2.5– 5.7) | 5.2 (3.4– 7.3) |
| 10 | — | — | 5.9 (4.6– 7.4) | 5.3 (3.9– 6.9) |
| 15 | — | — | 10.1 (9.1–11.1) | 11.3 (9.8–12.9) |
| 5 | MISO (5) | 5.7 (3.8– 7.8) | 7.6 (5.4–10.3) | 6.4 (4.7– 8.3) |
| 7.5 | MISO (5) | 6.4 (4.7– 8.4) | 11.1 (9.0–13.5) | 7.7 (6.0– 9.6) |
| 5 | 1902 (2.5) | 8.8 (6.6–11.5) | 6.9 (4.8– 9.4) | 4.4 (2.7– 6.4) |
| 7.5 | 1902 (2.5) | 10.3 (8.4–12.4) | 9.6 (7.9 –11.5) | 9.6 (8.3–10.9) |
| 5 | BENZO vehicle | 5.2 (3.5– 7.1) | 3.1 (1.3– 5.3) | 2.1 (1.1– 3.2) |
| 7.5 | BENZO vehicle | — | 7.8 (5.6–10.2) | 5.9 (4.7– 7.3) |
| 5 | BENZO (0.3) | 7.3 (4.1–11.3) | 6.3 (4.0– 9.0) | 4.3 (2.9– 6.0) |
| 7.5 | BENZO (0.3) | 6.0, 10.5, 13.0* | 7.1 (5.5– 8.9) | 6.3 (4.6– 8.3) |

*In this first experiment, BENZO was administered in a volume of $0.01\,ml\,g^{-1}$ and in this group, 7/10 mice died; the 3 values given are for the individual survivors. In the other experiments, BENZO was administered in a volume of $0.005\,ml\,g^{-1}$.

**Table V** Effect of MISO, 1902 amd BENZO on $LD_{50/30}$ for MEL

| *Experiment* | *Sensitizer and dose* ($mmol\,kg^{-1}$) | $LD_{50/30}$ *for MEL* ($mg\,kg^{-1}$) *(95% C.L.)* | *Dose modifying Factor (95% C.L.)* |
|---|---|---|---|
| A | — | 17.6 (14.8–20.9) | — |
| | MISO (5) | 9.2 (7.0–12.1) | 1.91 (1.38–2.64) |
| | 1902 (2.5) | 9.5 (6.9–13.1) | 1.85 (1.29–2.66) |
| | BENZO vehicle | 11.4 (4.8–27.4) | 1.54 (0.64–3.73) |
| | BENZO (0.3) | 9.5 (6.9–13.1) | 1.85 (1.35–2.55) |
| B | — | 22.0 (21.1–22.9) | — |
| | MISO (5) | 14.4 (6.9–30.3) | 1.53 (0.73–3.19) |
| | 1902 (2.5) | 12.5 (9.4–16.7) | 1.76 (1.32–2.35) |
| | BENZO vehicle | 12.7 (10.7–15.1) | 1.73 (1.45–2.07) |
| | BENZO (0.3) | 10.4 (7.9–13.6) | 2.12 (1.60–2.79) |
| A+B | — | 19.5 (17.6–21.6) | – |
| combined | MISO (5) | 11.4 (9.8–13.3) | 1.71 (1.42–2.05) |
| | 1902 (2.5) | 10.6 (9.5–11.9) | 1.84 (1.58–2.14) |
| | BENZO vehicle | 12.1 (10.9–13.1) | 1.61 (1.39–1.86) |
| | BENZO (0.3) | 10.0 (9.5–10.5) | 1.95 (1.74–2.18) |

Value of $LD_{50/30}$ determined on groups of 4 mice receiving graded doses of MEL and computed using the GLIM programme for probit analysis.

answer the question "which sensitizer produces the best therapeutic gain in combination with CCNU?" (i.e. MISO, 1902 or BENZO). We do not feel, however, that any one of them is clearly superior or inferior to the others. What we can say is that these effects can be produced at very low doses of BENZO. A similar conclusion has been reached by Siemann *et al.* (1983) based on their own data. We have shown (Workman & Twentyman, 1982) that enhancement of the response of the KHT tumour is maintained at BENZO doses down to $0.05\,mmol\,kg^{-1}$. This is similar to the dose levels which have been used in the treatment of Chaga's disease in man (Coura *et al.*, 1978; Raaflub, 1980)

**Figure 8** White cell count in tail vein blood measured 3 days after various doses of MEL. (△) Hanks BSS 30 min before MEL; (●) BENZO vehicle 30 min before MEL ;(○) BENZO (0.3 mmol kg$^{-1}$) 30 min before MEL.

and are currently being achieved in a phase 1 clinical study in this unit (Roberts *et al.*, unpublished). The balance of evidence from mouse studies is that enhancement of tumour response to CCNU is minimal below a MISO dose of 1.25 mmol kg$^{-1}$ (Workman & Twentyman, 1982; Hirst *et al.*, 1982) (although it should be noted that substantial enhancement at 1.25 mmol kg$^{-1}$ was seen by Siemann (1981)). This dose produces a peak plasma concentration in the mouse of 1.25 mM (Workman, 1980). The maximum dose of MISO which is generally given in the clinic (i.e. 3 g m$^{-2}$), however, only produces peak plasma levels of 0.5–0.75 mM (Workman, 1980). On this evidence, therefore, BENZO would appear to be a better candidate as an enhancer fo CCNU for clinical use.

There is, however, the additional factor that the elimination half-life of MISO is much longer in man than in the mouse, and this may, at least in part, compensate for lower peak levels. A number of studies have been, or are being, carried out to determine whether repeated administration of MISO to mice in order to maintain plasma levels at around 0.5 mM ($=100\,\mu$g ml$^{-1}$) can produce chemosensitization. Our own data (Twentyman & Workman, 1983) indicate minimal chemosensitization to CCNU, CTX or MEL by a 7 h regime of MISO administration, although a set of results showing greater sensitization has been reported by Brown and Hirst (1982). Our results of different time intervals between BENZO and CCNU reported here are relevant to this question. Following a dose of 2.5 mmol kg$^{-1}$ BENZO to the mouse, a plasma concentration of 0.46 mM is maintained between 2 h and 8 h followed by a fall to 0.06 mM at 16 h (Workman *et al.*, in preparation). At a dose of 0.3 mmol kg$^{-1}$, the peak of 0.15 mM is achieved within 30 min, with a subsequent elimination half life of 2.1 h, giving a concentration of 0.025 mM at 8 h and undetectable levels by 24 h (i.e. $<2\times10^{-4}$ mM).

Our finding that significant enhancement of CCNU in the KHT tumour remains at 28 h after 0.3 mmol kg$^{-1}$ BENZO indicates that the effect is not dependent upon the presence of significant sensitizer plasma levels. On the other hand, the finding that, for both 0.3 and 2.5 mmol kg$^{-1}$ of BENZO, sensitization is similar for CCNU administration at 30 min and 8 h after BENZO would indicate that length of pre-exposure to a given plasma level of BENZO is not the critical factor and our data for the multiple injection regime with BENZO support this conclusion. We have recently shown (Workman *et al.*, 1983) that MISO, 1902 and BENZO all act as inhibitors of drug metabolism and that these same agents cause major changes in the pharmacokinetics of CCNU and its metabolites (Lee & Workman, 1983 and in preparation). As far as MISO is concerned, the observed pharmacokinetic changes resulted in increased peak tumour concentrations of CCNU in the absence of any increase in peak concentrations in normal tissues, thus providing a likely basis for differential chemosensitization of the tumour (Lee & Workman, 1983 and in preparation). It seems likely that such pharmacokinetic changes are largely responsible for the modified tumour and normal tissue responses which we are now reporting for BENZO. Sensitization in the absence of detectable plasma concentrations of BENZO may be due to the presence of residual bound drug or metabolities in the liver or elsewhere, and we are currently carrying out experiments to investigate this.

Our results combining 1902 and BENZO with CTX indicate that these combinations are ineffective. The result for CTX+BENZO is in agreement with the data of McNally (private communication) for the WHT fibrosarcoma. These results may indicate that lipophilic sensitizers are able to interfere with CTX activation by liver microsomal enzymes as well as with cytotoxic drug detoxification. Combining the sensitizers with MEL certainly enhances the tumour response, in agreement with the observation of Sheldon & Batten (1982) that BENZO causes considerably greater enhancement of MEL response than does MISO in their MT tumour system. The very large increases in toxicity which we have seen, however, do not suggest that such combinations are likely to be of therapeutic value. There is a major problem, however, in combining BENZO with MEL in that the glycol vehicle used for BENZO does itself enhance MEL (but not CCNU) in both tumour response (Table IV) and particularly in terms of $LD_{50/30}$ (Table V). In these circumstances it is not possible to determine the extent to which sensitization to MEL would be brought about by BENZO in the absence of vehicle effects.

At the present time, it appears to us that the combination of CCNU with BENZO offers the most promise of possible clinical benefit based on current experimental data for chemosensitization by nitroimidazoles.

The nitroimidazoles used in these studies were kindly supplied by Dr. C.E. Smithen of Roche (Welwyn). We thank Jane Donaldson, Kate Smith and Michael Walton for their technical assistance.

### References

BROWN, J.M. & HIRST, D.G. (1982). The effect of clinically achievable exposure levels of misonidazole on the response of tumour and normal tissues in the mouse to alkylating agents. *Br. J. Cancer,* **45,** 700.

COURA, J.R., BR'NDEIRO, P.J. & FERREIRA, I. (1978). Benzidazole in the treatment of Chaga's disease. In *Current Chemotherapy* (Eds. Siegenthaler & Luthy). Washington: American Society for Microbiology. p. 161.

HIRST, D.G., BROWN, J.M. & HAZLEHURST, J.L. (1982). Enhancement of CCNU cytotoxicity by misonidazole: studies of the therapeutic ratio. *Br. J. Cancer,* **46,** 109.

KALLMAN, R.F., SILINI, G. & VAN PUTTEN, L.M. (1967). Factors influencing the quantitative estimation of the *in vivo* survival of cells from solid tumours. *J. Natl Cancer Inst.,* **39,** 539.

LEE, F.Y.F. & WORKMAN, P. (1983). Modification of CCNU pharmacokinetics by misonidazole—a major mechanism of chemosensitization in mice. *Br. J. Cancer,* **47,** 659.

RAAFLUB, J. (1980). Multiple-dose kinetics of trypanosomicide benznidazole in man. *Arzneim. Forsch.,* **30,** 2192.

SHELDON, P.W. & BATTEN, E.L. (1982). Potentiation *in vivo* of melphalan activity by nitroimidazole compounds. *Int. J. Radiat. Oncol. Biol. Phys.,* **8,** 635.

SIEMANN, D.W. (1981). The *in vivo* combination of the nitroimidazole misonidazole and the chemotherapeutic agent CCNU. *Br. J. Cancer,* **43,** 367.

SIEMANN, D.W. (1982). Response of murine tumours to combinations of CCNU, with misonidazole and other radiation sensitizers. *Br. J. Cancer,* **45,** 272.

SIEMANN, D.W., MORRISEY, S. & WOLF, K. (1983). The *in vivo* potentiation of CCNU by the radiation sensitizer benznidazole. *Cancer Res.,* **43,** 1010.

TWENTYMAN, P.R. (1981). Modification of tumour and host response to chemotherapy by misonidazole or by WR 2721. *Br. J. Radiol.,* **54,** 369.

TWENTYMAN, P.R., BROWN, J.M., GRAY, J.W., FRANKO, A.J., SCOLES, M.A. & KALLMAN, R.F. (1980). A new mouse tumour model system (RIF-1) for comparison of end-point studies. *J. Natl Cancer Inst.,* **64,** 595.

TWENTYMAN, P.R., KALLMAN, R.F. & BROWN, J.M. (1979). The effect of time between X-irradiation and chemotherapy on the growth of three solid mouse tumours: I. Adriamycin. *Int. J. Radiat. Oncol. Biol. Phys.,* **5,** 1255.

TWENTYMAN, P.R. & WORKMAN, P. (1982). Effect of misonidazole or metronidazole pretreatment on the response of the RIF-1 mouse sacroma to melphalan, cyclophosphamide, chlorambucil and CCNU. *Br. J. Cancer,* **45,** 447.

TWENTYMAN, P.R. & WORKMAN, P. (1983). An investigation of the possibility of chemosensitization by clinically achievable concentrations of misonidazole. *Br. J. Cancer,* **47,** 187.

WORKMAN, P. (1980). Pharmacokinetics of hypoxic cell radiosensitizers. A review. *Cancer Clin. Trials,* **3,** 237.

WORKMAN, P., LITTLE, C.J., MARTEN, T.R. & 4 others. (1978). Estimation of the hypoxic cell-sensitizer misonidazole and its O-demethylated metabolite in biological materials by reversed-phase high-performance liquid chromatography. *J. Chromatog.,* **145,** 507.

WORKMAN, P., TWENTYMAN, P.R., LEE, F.Y.F. & WALTON, M.I. (1983). Drug metabolism and chemosensitization: nitroimidazoles as inhibitors of drug metabolism. *Biochem. Pharmacol.,* **32,** 857.

WORKMAN, P. & TWENTYMAN, P.R. (1982). Structure-activity relationships for the enhancement by electron-affinic drugs of the anti-tumour effect of the nitrosourea, CCNU. *Br. J. Cancer,* **46,** 249.